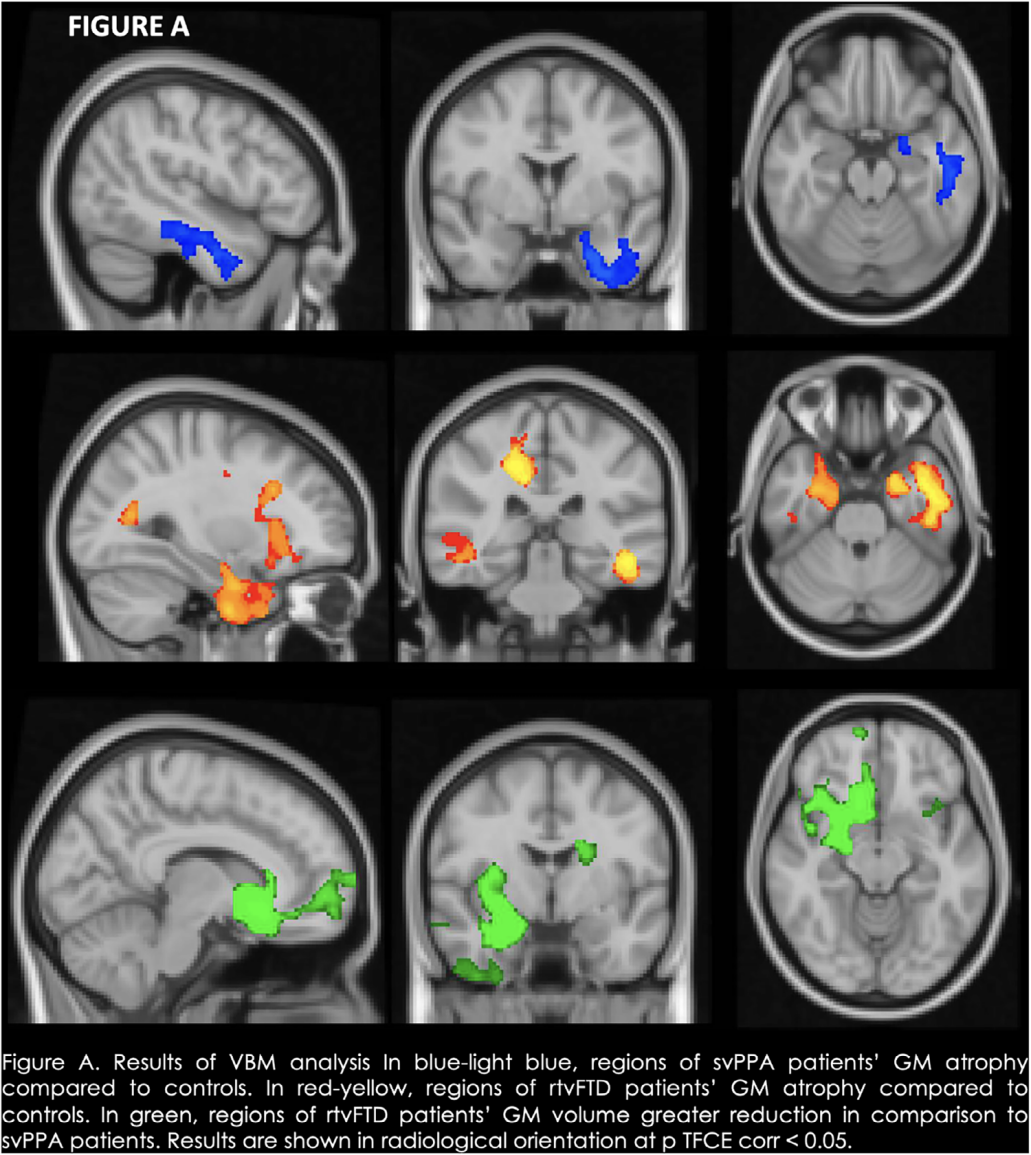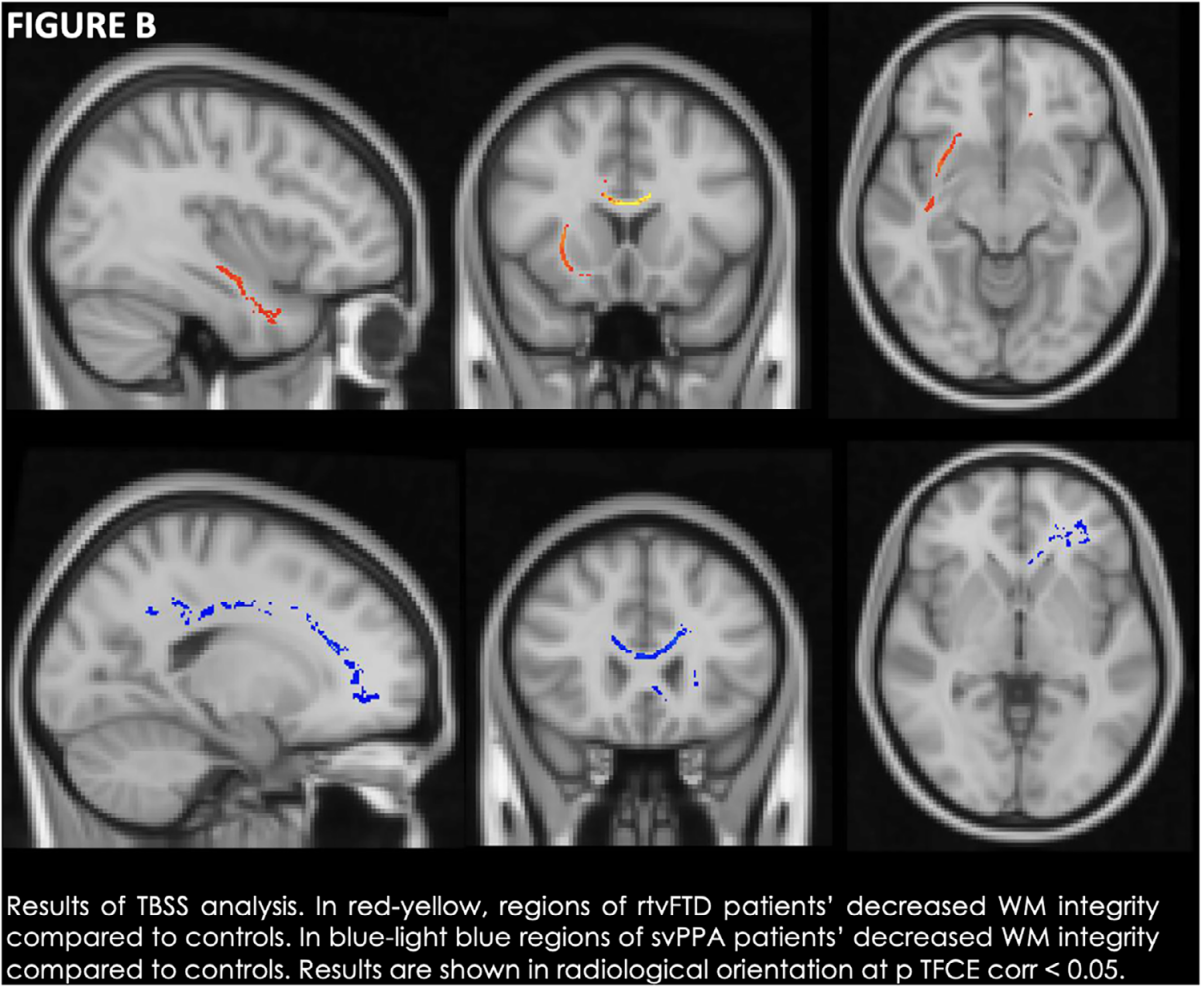# Right temporal variant frontotemporal dementia: a multimodal MRI analysis

**DOI:** 10.1002/alz.090317

**Published:** 2025-01-09

**Authors:** Chiara Gallingani, Chiara Carbone, Davide Salvatori, Manuela Tondelli, Giulia Vinceti, Annalisa Chiari, Giovanna Zamboni

**Affiliations:** ^1^ Università di Modena e Reggio Emilia, Modena Italy; ^2^ Neurologia, Azienda Ospedaliero Universitaria di Modena, Modena Italy

## Abstract

**Background:**

Right temporal variant frontotemporal dementia (rtvFTD), a new recognized entity among the FTD‐spectrum, is characterized by right anterior temporal lobe (rATL) atrophy and a peculiar clinical presentation, involving face and emotions recognition, memory, and naming deficits and behavioral disturbances. Clinical diagnosis is challenging, since rtvFTD shares features with both the behavioral variant FTD (bvFTD) and the semantic variant primary progressive aphasia (svPPA), and there is no consensus yet on its designation and characterization. Although rATL neurodegeneration is a hallmark of this syndrome, only a few studies investigated patterns of gray matter (GM) atrophy. Even less is known about white matter (WM) involvement. We conducted a preliminary study on the rtvFTD neuroimaging features using multimodal magnetic resonance imaging (MRI).

**Method:**

We compared GM volume and WM microstructural integrity in rtvFTD (n=3) and svPPA (n=3) patients, and healthy controls (HC, n=27), using voxel‐based morphometry (VBM) and tract‐based spatial statistics (TBSS). Age and disease duration were considered as covariates of no interest.

**Result:**

SvPPA patients showed GM atrophy in the left temporal structures relative to HC. rtvFTD patients compared to HC showed not only GM atrophy in the right frontal and temporal structures, but also in the insula bilaterally, and in the left temporal and orbitofrontal cortices. Direct comparison between rtvFTD and svPPA showed that rtvFTD were more atrophic than svPPA patients in the bilateral temporal and frontal regions. Moreover, rtvFTD patients had less WM integrity than controls in the corpus callosum and the right inferior fronto‐occipital, inferior longitudinal, and uncinate fasciculi, while svPPA patients had decreased WM integrity in the corpus callosum and the left inferior fronto‐occipital fasciculus.

**Conclusion:**

We found that rtvFTD patients show greater atrophy compared to svPPA patients both in the right and left hemisphere, independently of disease duration. This suggests that rtvFTD and svPPA do not mirror each other in GM loss, and that a neurodegenerative process starting in the right hemisphere must be more widespread to become clinically evident. We also demonstrate that rtvFTD patients show WM disruption in fasciculi which have been implicated in face recognition, emotion processing, and language functions, in line with the clinical picture.